# Effect of centre volume on pathological outcomes and postoperative complications after surgery for colorectal cancer: results of a multicentre national study

**DOI:** 10.1093/bjs/znad373

**Published:** 2023-11-14

**Authors:** Matteo Rottoli, Antonino Spinelli, Gianluca Pellino, Alice Gori, Giacomo Calini, Maria E Flacco, Lamberto Manzoli, Gilberto Poggioli, the COVID-CRC Study Group, Angela Romano, Angela Belvedere, Antonio Lanci Lanci, Daniele Parlanti, Gabriele Vago, Anna Paola Pezzuto, Anna Canavese, Gerti Dajti, Stefano Cardelli, Caterina Catalioto, Iris S Russo, Tommaso Violante, Daniele Morezzi, Ludovica Maurino, Eleonora Filippone, Dajana Cuicchi, Paolo Bernante, Elio Jovine, Raffaele Lombardi, Michele Masetti, Chiara Cipressi, Maria F Offi, Cristina Larotonda, Silvana B Puglisi, Augusto Barbosa, Roberto Vaiana, Paolo M Bianchi, Carlo Tonti, Claudio Codignola, Luigi Zorcolo, Angelo Restivo, Simona Deidda, Marcello E Marchetti, Luca Ippolito, Gaya Spolverato, Salvatore Pucciarelli, Francesco Marchegiani, Giacomo Ghio, Gaia Zagolin, Dajana Glavas, Monica Tomassi, Riccardo Rosati, Ugo Elmore, Lorenzo Gozzini, Riccardo Calef, Francesco Puccetti, Andrea Cossu, Andrea Vignali, Mario Morino, Marco E Allaix, Gaspare Cannata, Erica Lombardi, Carlo A Ammirati, Chiara Piceni, Piero Buccianti, Riccardo Balestri, Marco Puccini, Daniele Pezzati, Roberto d'Ischia, Vito F Asta, Benedetta Sargenti, Giacomo Taddei, Federica Bonari, Giulia Boni, Alessandro Ferrero, Michela Mineccia, Federica Gonella, Marco Palisi, Francesco Danese, Valeria Cherubini, Serena Perotti, Michele Carvello, Fabio Carbone, Antonio Luberto, Eleonora Calafiore, Francesca De Lucia, Matteo Sacchi, Diego Sasia, Maria C Giuffrida, Edoardo Ballauri, Mathieu Cardile, Serena Armentano, Elsa Beltrami, Gabriele Preve, Barbara Vercellone, Marta Mozzon, Cristina Folliero, Chiara Lirusso, Massimo Vecchiato, Antonio Ziccarelli, Davide Gattesco, Luisa Moretti, Sara Crestale, Filippo Banchini, Patrizio Capelli, Andrea Romboli, Gerardo Palmieri, Luigi Conti, Nicholas Rizzi, Deborah Bonfili, Nicolò de Manzini, Paola Germani, Edoardo Osenda, Sara Cortinovis, Carlotta Giunta, Stefano Fracon, Hussein Abdallah, Selene Bogoni, Nazario Portolani, Riccardo Nascimbeni, Sarah Molfino, Guido A M Tiberio, Ilenia Garosio, Giulia Lamperti, Diego Rigosa, Giorgio Ercolani, Leonardo Solaini, Davide Cavaliere, Andrea Avanzolini, Fabrizio D'Acapito, Leonardo L Chiarella, Daniela Di Pietrantonio, Domenico Annunziata, Roberta Piccolo, Mario Sorrentino, Mauro Pansini, Alessandro Cojutti, Michele Graziano, Francesco Callegari, Laura Balzarotti, Vitale R Dameno, Antonio Cattaneo, Giuliano Santolamazza, Caterina Altieri, Riccardo Magarini, Andrea Pietrabissa, Tommaso Dominioni, Luigi Pugliese, Andrea Peri, Marta Botti, Benedetta Sargenti, Francesco Salvetti, Luigi Boni, Elisa Cassinotti, Ludovica Baldari, Valentina Messina, Vera D'Abrosca, Pasquale Cianci, Rocco Tumolo, Domenico Gattulli, Enrico Restini, Marina Minafra, Maria Grazia Sederino, Bernardino Bottalico, Pierluigi Pilati, Boris Franzato, Genny Mattara, Ottavia De Simoni, Andrea Barina, Marco Tonello, Andrea Muratore, Marcello Calabrò, Nicoletta S Federico Pipitone, Bruno Cuzzola, Elena Herranz Van Nood, Nicola Passuello, Alvise Frasson, Enzo Mammano, Luca Faccio, Fabrizio Vittadello, Alice Bressan, Giacomo Sarzo, Nicolò Tamini, Massimo Oldani, Luca Cigagna, Francesca Carissimi, Giulia De Carlo, Edoardo Baccalini, Luca Nespoli, Alessio Giordano, Stefano Cantafio, Lucrezia Grifoni, Davide Matani, Serena Livi, Daniele Delogu, Fabrizio Scognamillo, Antonio Marrosu, Luca Guerrini, Giampaolo Ugolini, Federico Ghignone, Giacomo Frascaroli, Nicola Albertini, Davide Zattoni, Giovanni Taffurelli, Isacco Montroni, Francesco Colombo, Piergiorgio Danelli, Andrea Bondurri, Anna Maffioli, Alessandro Bonomi, Isabella Pezzoli, Francesco Cammarata, Orlando Goletti, Mattia Molteni, Alberto Assisi, Giorgio Quartierini, Corrado Da Lio, Daunia Verdi, Isabella Mondi, Claudia Peluso, Lorenzo Macchi, Marta Tanzanu, Federico Zanzi, Sara Pellegrini, Jacopo Andreuccetti, Rossella D'Alessio, Giusto Pignata, Michele De Capua, Ilaria Canfora, Luca Ottaviani, Pasquale Lepiane, Andrea Balla, Antonio De Carlo, Federica Saraceno, Rosa Scaramuzzo, Anna Guida, Daniele Aguzzi, Paolo Bellora, Sergio Gentilli, Manuela Monni, Herald Nikaj, Nicola Cillara, Alessandro Cannavera, Antonello Deserra, Carla Margiani, Roberta Cabula, Manuela Dettori, Giulia Gramignano, Giovanni Lezoche, Monica Ortenzi, Elena S Orlandoni, Federica Curzi, Francesca Vitali, Perla Capomagi, Miriam Palmieri, Mario Giuffrida, Paolo Del Rio, Elena Bonati, Tommaso Loderer, Federico Cozzani, Matteo Rossini, Stefano Agnesi, Gabriella T Capolupo, Marco Caricato, Filippo Carannante, Gianluca Mascianà, Martina Marrelli, Valentina Miacci, Sara Lauricella, Valeria Tonini, Maurizio Cervellera, Salvatore Pisconti, Concetta Lozito, Juliana Shahu, Claudia Mongelli, Giulia Morelli, Lodovico Sartarelli, Giuseppe S Sica, Leandro Siragusa, Giulia Bagaglini, Andrea M Guida, Marzia Franceschilli, Vittoria Bellato, Cristina Fiorani, Antonio Taddei, Matteo Risaliti, Ilenia Bartolini, Maria N Ringressi, Luca Tirloni, Letizia Laface, Emmanuele Abate, Massimiliano Casati, Pietro Gobbi, Enrico Opocher, Nicolò M Mariani, Andrea Pisani Ceretti, Marco Giovenzana, Beatrice Giuliani, Martina Sironi, Ugo Grossi, Giacomo Zanus, Giulio Aniello Santoro, Marco Brizzolari, Eugenio De Leo, Simone Novello, Krizia Aquilino, Francesco Milardi, Stefano Olmi, Matteo Uccelli, Marta Bonaldi, Giovanni C Cesana, Marco Bindi, Raffaele Galleano, Antonio Langone, Massimiliano Botto, Angelo Franceschi, Elena Gambino, Maurizio Ronconi, Silvia Casiraghi, Giovanni Casole, Salvatore L Ciulla, Giovanni Terrosu, Sergio Calandra, Edoardo Scarpa, Vittorio Cherchi, Giacomo Calini, Lisa Martinuzzo, Lucrezia Clocchiatti, Davide Muschitiello, Andrea Romanzi, Barbara Vignati, Alberto Vannelli, Roberta Scolaro, Maria Milanesi, Fabrizio Rossi, Giuseppe Canonico, Alessandro Anastasi, Tommaso Nelli, Marco Barlettai, Riccardo Fratarcangeli, Carmela Di Martino, Andrea Damigella, Elvira Adinolfi, Arianna Birindelli, Lucio Taglietti, Sara E Dester, Francesco Fleres, Eugenio Cucinotta, Francesca Viscosi, Santino A Biondo, Giorgio Badessi, Nivia Catarsini, Carmelo Mazzeo, Daniela Rega, Paolo Delrio, Carmela Cervone, Alessia Aversano, Silvia De Franciscis, Massimiliano Di Marzo, Bruno Marra, Ugo Pace, Antonio Amato, Paola Batistotti, Elisa Mina, Alberto Serventi, Pierfrancesco Lapolla, Andrea Mingoli, Paolo Sapienza, Gioia Brachini, Bruno Cirillo, Enrico Fiori, Daniele Crocetti, Ilaria Clementi, Gennaro Martines, Arcangelo Picciariello, Giovanni Tomasicchio, Rigers Dibra, Giuseppe Trigiante, Marcella Rinaldi, Giuliano Lantone, Alberto Porcu, Teresa Perra, Antonio M Scanu, Claudio F Feo, Alessandro Fancellu, Maria L Cossu, Giorgio C Ginesu, Alberto Patriti, Diego Coletta, Filippo Petrelli, Paola A Greco, Claudia Spadoni, Giovanna Cassiani, Federica Bianchini, Marco Arganini, Matteo Bianchini, Bruno Perotti, Matteo Palmeri, Stefano Scabini, Selene Deiana, Giacomo Carganico, Davide Pertile, Domenico Soriero, Emanuela Fioravanti, Beatrice Sperotto, Bruno Nardo, Daniele Paglione, Veronica Crocco, Marco Doni, Mariasara Osso, Roberto Perri, Gianluca M Sampietro, Carlo Corbellini, Leonardo Lorusso, Carlo A Manzo, Maria Cigognini, Caterina Baldi, Giuseppe Palomba, Giovanni Aprea, Marianna Capuano, Raffaele Basile, Roberta Tutino, Marco Massani, Laura Marinelli, Nicola Canitano, Tiziana Pilia, Mauro Podda, Adolfo Pisanu, Valentina Murzi, Silvia Incani, Federica Frongia, Giuseppe Esposito, Gaetano Luglio, Francesca P Tropeano, Gianluca Pagano, Eduardo Spina, Giuseppe De Simone, Michele Cricrì, Fausto Catena, Carlo Vallicelli, Nicola Zanini, Diana Ronconi, Francesco Favi, Carlo Mazzucchelli, Girolamo Convertini, Leonardo Vincenti, Valeria Andriola, Cinzia Bizzoca, Carlo V Feo, Nicolò Fabbri, Marta Fazzin, Antonio Pesce, Silvia Gennari, Marco Torchiaro, Silvia Severi, Alice Frontali, Greta Bracchetti, Stefano Granieri, Christian Cotsoglou, Massimo Carlini, Giorgio Lisi, Domenico Spoletini, Maria R Mastrangeli, Michela Campanelli, Michele Manigrasso, Marco Milone, Giovanni D De Palma, Sara Vertaldi, Alessia Chini, Francesco Maione, Alessandra Marello, Francesco Selvaggi, Guido Sciaudone, Lucio Selvaggi, Francesco Menegon Tasselli, Giacomo Fuschillo, Lidia Oddis, Simona Grande, Michele Grande, Simona Ascanelli, Laura Chimisso, Filippo Aisoni, Eleonora Rossin, Francesco Pepe, Francesco Marchetti, Biagio Picardi, Stefano Rossi, Simone Rossi Del Monte, Matteo Picarelli, Irnerio A Muttillo, Carlo Ratto, Angelo A Marra, Angelo Parello, Francesco Litta, Paola Campennì, Veronica De Simone, Francesco Pata, Cristiana Riboni, Emanuele Rausa, Valerio Celentano

**Affiliations:** Surgery of the Alimentary Tract, IRCCS Azienda Ospedaliero-Universitaria di Bologna, Bologna, Italy; Department of Medical and Surgical Sciences, Alma Mater Studiorum University of Bologna, Bologna, Italy; Department of Biomedical Sciences, Humanitas University, Milan, Italy; Colorectal Surgery, RCCS Humanitas Research Hospital, Milan, Italy; Department of Advanced Medical and Surgical Sciences, Università degli Studi della Campania Luigi Vanvitelli, Naples, Italy; Colorectal Surgery, University Hospital Vall d’Hebron, Barcelona, Spain; Surgery of the Alimentary Tract, IRCCS Azienda Ospedaliero-Universitaria di Bologna, Bologna, Italy; Department of Medical and Surgical Sciences, Alma Mater Studiorum University of Bologna, Bologna, Italy; Surgery of the Alimentary Tract, IRCCS Azienda Ospedaliero-Universitaria di Bologna, Bologna, Italy; Department of Medical and Surgical Sciences, Alma Mater Studiorum University of Bologna, Bologna, Italy; Department of Environmental and Preventive Sciences, University of Ferrara, Ferrara, Italy; Department of Medical and Surgical Sciences, Alma Mater Studiorum University of Bologna, Bologna, Italy; Surgery of the Alimentary Tract, IRCCS Azienda Ospedaliero-Universitaria di Bologna, Bologna, Italy; Department of Medical and Surgical Sciences, Alma Mater Studiorum University of Bologna, Bologna, Italy

## Abstract

**Background:**

The association between volume, complications and pathological outcomes is still under debate regarding colorectal cancer surgery. The aim of the study was to assess the association between centre volume and severe complications, mortality, less-than-radical oncologic surgery, and indications for neoadjuvant therapy.

**Methods:**

Retrospective analysis of 16,883 colorectal cancer cases from 80 centres (2018–2021). Outcomes: 30-day mortality; Clavien-Dindo grade >2 complications; removal of ≥ 12 lymph nodes; non-radical resection; neoadjuvant therapy. Quartiles of hospital volumes were classified as LOW, MEDIUM, HIGH, and VERY HIGH. Independent predictors, both overall and for rectal cancer, were evaluated using logistic regression including age, gender, AJCC stage and cancer site.

**Results:**

LOW-volume centres reported a higher rate of severe postoperative complications (OR 1.50, 95% c.i. 1.15–1.096, *P* = 0.003). The rate of ≥ 12 lymph nodes removed in LOW-volume (OR 0.68, 95% c.i. 0.56–0.85, *P* < 0.001) and MEDIUM-volume (OR 0.72, 95% c.i. 0.62–0.83, *P* < 0.001) centres was lower than in VERY HIGH-volume centres. Of the 4676 rectal cancer patients, the rate of ≥ 12 lymph nodes removed was lower in LOW-volume than in VERY HIGH-volume centres (OR 0.57, 95% c.i. 0.41–0.80, *P* = 0.001). A lower rate of neoadjuvant chemoradiation was associated with HIGH (OR 0.66, 95% c.i. 0.56–0.77, *P* < 0.001), MEDIUM (OR 0.75, 95% c.i. 0.60–0.92, *P* = 0.006), and LOW (OR 0.70, 95% c.i. 0.52–0.94, *P* = 0.019) volume centres (vs. VERY HIGH).

**Conclusion:**

Colorectal cancer surgery in low-volume centres is at higher risk of suboptimal management, poor postoperative outcomes, and less-than-adequate oncologic resections. Centralisation of rectal cancer cases should be taken into consideration to optimise the outcomes.

## Introduction

A conclusive association between low case volume and poor postoperative and oncological outcomes has yet to be defined in the setting of colorectal cancer surgery. While several studies have suggested that specific perioperative outcomes would improve with increased caseload^1^, others have not reported any volume–outcome relationship^2^. Furthermore, identifying these outcomes and the volume thresholds has proven to be unreliable^3^. The great variations among the different studies are most likely due to different settings and study populations. For instance, the use of administrative data sets, which have the advantage of providing a large number of cases, reduces the possibility of adjusting the analyses for the many confounders that should be taken into account regarding their effect on outcomes^4,5^. On the other hand, collaborative research studies often include a limited number of cases per centre and a higher proportion of high-volume centres^6,7^.

The aim of the present study was to analyse the data from a large collaborative study that included high- and low-volume centres over a four-year period, in order to identify potential correlations between hospital volume, mortality, postoperative complications and oncological outcomes.

## Methods

The coronavirus disease 2019 (COVID-19) ColoRectal Cancer (COVID-CRC) Collaborative study data set retrospectively included 17 938 patients undergoing surgery for colorectal cancer between January 2018 and December 2021 in 80 Italian hospitals^8^. No minimum number of cases was required for the centres to register for the study; thus tertiary centres and community hospitals were enrolled regardless of hospital volume.

The variables of interest were identified using institutional databases and entered into a REDCap (Research Electronic Data Capture)^9^ database by a team of clinicians. A proportion of cases (20%) was subsequently validated by an independent investigator in each centre.

The data set reported the details of co-morbidities, preoperative diagnosis, neoadjuvant therapy, surgery (type of operation and intraoperative complications) and postoperative outcome at 30 days from surgery. The histological details included the stage and the biological characteristics of the tumour, as well as the radicality of surgery.

The following inclusion criteria were adopted: patients ≥ 18 years of age; any type of colorectal surgery for cancer (including surgery after non-radical endoscopic excision of a cancer); elective or urgent surgery with curative intent; location of cancer in the colon, rectum or anus; and a minimum follow-up of 30 days from surgery. The exclusion criteria were recurrent cancer and cancer originating from other locations, cancers other than adenocarcinoma or squamous cell carcinoma, benign lesions and palliative surgery (defined as procedures not aiming to radically remove the primary tumour).

The main aim of the study was to assess the correlation among different volumes of procedures and the following five outcomes: 30-day mortality, severe postoperative complications (defined according to the Clavien–Dindo classification > grade 2)^10^, adequate lymph node sampling (≥12 lymph nodes on the histological specimen), non-radical resection (defined as involvement of any margin from the tumour) and use of neoadjuvant therapy. A subgroup analysis was carried out including only patients undergoing surgery for rectal cancer.

The Ethics Committees of all participating centres approved the study, which was registered on ClinicalTrials.gov (NCT04712292). A written consent form was obtained from all available patients. The study followed the STROBE guidelines^11^.

## Statistical analysis

Hospitals were ranked by volume according to the number of interventions performed between 2018 and 2021. In order to evaluate the association between all variables recorded and hospital procedure volumes, quartiles of hospital volumes based on the number of interventions performed in the four-year study period were identified, namely low (first quartile—between 19 and 111 procedures), medium (second quartile—between 112 and 167 procedures), high (third quartile—168 and 263 procedures) and very high (fourth quartile— ≥ 264 procedures). Potential differences in the distribution of all clinical characteristics recorded across quartiles of hospital volume were then assessed using the chi-squared test for categorical variables, and one-way ANOVA with Bonferroni correction for continuous variables.

For each of the five outcomes of interest, potential independent predictors were separately evaluated using logistic regression. The covariates were tested for multi-collinearity and selected for inclusion in the final models using a stepwise forward process with the following inclusion criteria: clinical relevance, *P* < 0.10 at univariate analyses, age, gender, AJCC stage, cancer site (rectum *versus* others), and urgent surgery. As a separate, additional analysis, the same outcomes were evaluated in the subsample of patients undergoing surgery for rectal cancer.

For the latter analyses, hospital volume was categorized both using quartiles and adopting a minimum threshold of ≥10 surgical procedures per year, in accordance with the National Institute for Health and Care Excellence (NICE) guidelines^12^. Accordingly, all analyses were repeated comparing low-volume (<10 surgical procedures per year) *versus* high-volume (≥10 per year) centres. Standard diagnostic procedures were adopted to check the validity of all the models, including influential observation analysis (Dbeta, change in Pearson chi-square), Hosmer–Lemeshow test for the goodness of fit, and C statistic (area under the receiving operator characteristic curve). Statistical significance was defined as a two-sided *P* < 0.05. All analyses were carried out using Stata, version 13.1 (Stata Corp., College Station, Texas, 2014).

## Results

A total of 16 883 patients treated in 80 centres were included in the final analysis. *Tables 1*, *2* show the distribution of the demographic and clinical characteristics, as well as the five outcomes using the quartiles of hospital volume (low volume: 21 centres, 999 patients; medium volume: 19 centres, 2644 patients; high volume: 20 centres, 4675 patients; very high volume: 20 centres, 8565 patients). *Tables 1*, *2* also report the univariate comparisons among the quartiles. A significantly higher rate of cancer located in the rectum was reported in the very high-volume centres (30.4%) as compared to the high- (24.6%), medium- (25.9%) and low-volume (23.4%) centres. Further detail of centre volume is shown in *Table S1*.

**Table 1:** Selected demographic and clinical characteristics, and outcomes for the whole cohort and stratified by hospital volume

	Overall sample	Patients by hospital volume*				*P*#
		Low†	Medium‡	High§	Very high¶	
Patients (hospitals), *n*	16 883 (80)	999 (21)	2644 (19)	4675 (20)	8565 (20)	
Male/female gender, %	44.1/55.9	44.9/55.1	45.4/54.6	44.6/55.4	43.4/56.6	
Mean age in years (s.d.)	70.4 (12.2)	70.4 (12.2)	71.3 (11.8)	70.8 (12.0)	69.8 (12.4)	^e, f^
**Co-morbidities, %**						
None	23.5	23.6	22.1	25.8	22.6	^c, d, f^
1	39.9	36.6	42.9	36.6	41.2	^b, c, d, f^
2	24.5	24.8	23.8	25.1	24.4	^b, c, e^
3 or more	12.1	15.0	11.2	12.5	11.8	^a^
**Anatomical location, %**						
Right-transverse colon	42.7	42.7	45.3	43.8	41.4	^e, f^
Left colon	29.0	32.8	28.5	31.1	27.5	^a, c, d, f^
Rectum	27.7	23.4	25.9	24.6	30.4	^c, e, f^
Multiple lesions	0.6	1.1	0.3	0.5	0.7	^a, e^
T4 stage, %	6.0	8.7	6.2	3.8	6.9	^a, b, c, d, f^
*Rectal lesions only, %*	(*n* = 4676)	(*n* = 233)	(*n* = 685)	(*n* = 1153)	(*n* = 2605)	
N1 stage	40.0	37.8	35.2	33.9	44.2	^e, f^
T4 stage	6.9	8.2	4.8	6.6	7.5	^e^
Metastatic lesions, %	9.6	10.9	10.4	6.5	10.8	^b, d, f^
Stenosing lesions, %	13.1	14.8	11.4	11.0	14.6	^a, b, e, f^
Urgent surgery, %	8.8	17.5	11.4	7.6	7.6	^a, c, d, e^
ASA score >2, %	43.5	47.5	40.2	40.9	45.4	^a, b, e, f^
	(*N* = 12 722)	(*N* = 824)	(*N* = 2241)	(*N* = 3808)	(*N* = 5849)	
Anastomosis, %	93.2	89.1	93.4	92.7	93.9	^a, b, c, f^
Additional surgery, %	10.3	11.4	9.7	6.8	12.3	^b, d, e, f^
Laparoscopic surgery, %	74.5	58.1	67.7	76.8	77.2	^a, b, c, d, e^
	(*N* = 12 572)	(*N* = 580)	(*N* = 1789)	(*N* = 3592)	(*N* = 6611)	
Conversion to open surgery, %	7.3	8.3	8.1	6.1	7.6	^b, d, f^
	(*N* = 14 943)	(*N* = 868)	(*N* = 2372)	(*N* = 4228)	(*N* = 7475)	
Loop ileostomy, %	16.1	12.3	14.4	12.5	19.1	^c, d, e, f^
ICU admission, %	15.0	15.7	12.9	15.3	15.5	^a, d, e^

*Hospital procedure volume was defined as the number of operations performed during the study period (2018–2021). The unit of analysis was individual patients unless otherwise stated. †Low-volume hospitals performed between 19 and 111 procedures. ‡Medium-volume hospitals performed between 112 and 167 procedures. §High-volume hospitals performed between 168 and 263 procedures. ¶Very high-volume hospitals performed between 264 and 638 procedures. #Chi-squared test for the categorical variables; one-way ANOVA with Bonferroni corrections for continuous ones. a = *P* < 0.05 for the comparison between low- and medium-volume hospitals; b = *P* < 0.05 for the comparison between low- and high-volume hospitals; c = *P* < 0.05 for the comparison between low- and very high-volume hospitals; d = *P* < 0.05 for the comparison between medium- and high-volume hospitals; e = *P* < 0.05 for the comparison between medium- and very high-volume hospitals; f = *P* < 0.05 for the comparison between high- and very high-volume hospitals. All *P*s not indicated were >0.05.

**Table 2:** Pathological and postoperative variables and outcomes, overall and by hospital surgical volume

	Overall sample	Patients by hospital volume*				*P*#
		Low†	Medium‡	High§	Very high¶	
Patients (hospitals), *n*	16 883 (80)	999 (21)	2644 (19)	4675 (20)	8565 (20)	
**AJCC stage, %**	(*N* = 16 273)	(*N* = 979)	(*N* = 2526)	(*N* = 4535)	(*N* = 8233)	
0	3.2	3.6	3.4	3.3	3.1	
I	24.5	21.6	23.3	24.6	25.2	^b, c, e^
II	32.3	32.0	31.9	35.0	31.0	^c, f^
III	30.0	31.7	30.5	30.4	29.4	
IV	10.0	11.1	10.9	6.7	11.3	^c, e, f^
Median hospital length of stay (i.q.r.)	7.0 (5.0)	8.0 (5.0)	7.0 (4.0)	7.0 (4.0)	7.0 (5.0)	^a, b, e, f^
**Postoperative medical complications, %**						
All complications**	16.0	15.4	16.2	13.8	17.2	^d, f^
Anaemia	4.6	4.7	6.7	4.5	4.0	
Pulmonary complications††	3.1	5.0	2.3	3.3	3.0	
Sepsis	2.0	2.3	1.3	1.4	2.4	
Acute kidney failure	1.2	2.0	1.1	0.8	1.3	
Myocardial infarction	0.3	0.4	0.5	0.3	0.2	
Venous thromboembolism	0.2	0.0	0.2	0.1	0.3	
Pulmonary embolism	0.3	0.5	0.2	0.2	0.3	
Stroke	0.1	0.3	0.1	0.1	0.2	
**Postoperative surgical complications, %**						
All complications‡‡	17.7	19.5	15.0	14.9	19.9	^a, b, e, f^
Surgical site infection	4.0	5.8	3.6	3.0	4.4	
Intra-abdominal bleeding	1.1	1.9	1.1	1.1	1.0	
Intraluminal bleeding	1.2	1.1	0.7	1.4	1.2	
Intra-abdominal sepsis§§	7.1	7.6	5.5	6.0	8.1	
Dehiscence	5.0	4.5	4.2	4.6	5.6	
Abdominal abscess	2.6	3.4	1.5	1.7	3.5	
Peritonitis	0.9	1.1	0.3	0.7	1.1	
Paralytic ileus	2.9	1.5	2.3	2.6	3.4	
Bowel occlusion	1.0	1.4	0.8	0.8	1.1	
**Outcomes**						
30-day mortality, %	1.6	2.5	1.9	1.5	1.5	^b, c^
	(*N* = 4840)	(*N* = 289)	(*N* = 703)	(*N* = 1135)	(*N* = 2713)	
Clavien–Dindo classification ≥3, %	34.5	42.6	34.4	35.3	33.3	^a, b, c^
≥12 lymph nodes, %	85.2	82.6	83.3	86.7	85.4	^b, c, d, e, f^
Non-radical surgery (R1/2), %	2.6	2.0	2.2	2.3	2.9	^e^
Neoadjuvant therapy, %	15.4	11.6	12.3	11.6	18.8	^c, e, f^

*Hospital procedure volume was defined as the number of operations performed during the study period (2018–2021). The unit of analysis was individual patients unless otherwise stated. †Low-volume hospitals performed between 19 and 111 procedures. ‡Medium-volume hospitals performed between 112 and 167 procedures. §High-volume hospitals performed between 168 and 263 procedures. ¶Very high-volume hospitals performed between 264 and 638 procedures. #Chi-squared test for the categorical variables; one-way ANOVA with Bonferroni corrections for continuous ones. a = *P* < 0.05 for the comparison between low- and medium-volume hospitals; b = *P* < 0.05 for the comparison between low- and high-volume hospitals; c = *P* < 0.05 for the comparison between low- and very high-volume hospitals; d = *P* < 0.05 for the comparison between medium- and high-volume hospitals; e = *P* < 0.05 for the comparison between medium- and very high-volume hospitals; f = *P* < 0.05 for the comparison between high- and very high-volume hospitals. All the *P*s not indicated were >0.05. **Including: anaemia, myocardial infarction, stroke, pulmonary embolism, venous thromboembolism, acute kidney failure, sepsis, pneumonia, acute respiratory distress syndrome, respiratory failure. ††Pneumonia, and/or acute respiratory distress syndrome, and/or respiratory failure. ‡‡Including intra-abdominal or intraluminal bleeding, dehiscence, surgical site infection, abdominal abscess, peritonitis, paralytic ileus, bowel occlusion. §§Including dehiscence, abdominal abscess, peritonitis.

The rate of urgent surgery (defined as the need for surgery within 48 h of hospital admission) was significantly higher in the low-volume centres (17.5%) than in the medium- (11.4%), high- (7.6%) and very high-volume (7.6%) centres, while the use of a minimally invasive approach was significantly lower (58.1% *versus* 67.7%, 76.8% and 77.2%, respectively).

A significantly higher risk of mortality was found in the low-volume centres (2.5%) as compared to the other centres (1.9%, 1.5% and 1.5%, respectively). The risk of severe postoperative complications was also significantly higher in the low-volume centres (42.6%) than in the medium- (34.4%), high- (35.3%) and very high-volume (33.3%) centres.

The probability of a final AJCC stage I increased progressively from low-volume centres (21.6%) to very high-volume centres (25.2%). The proportion of cases with ≥ 12 lymph nodes removed during surgery was 82.6% in the low-volume centres, and significantly lower than that in the high- (86.7%) and very high-volume (85.4%) centres.

*Table 3* shows the multivariate analyses assessing the association between the quartile of case-volume and the five outcomes in the overall population, adjusted for age, gender, AJCC stage, cancer site (rectum *versus* others) and urgent surgery. The low-volume centres were significantly associated with a higher risk of severe complications (OR 1.5, 95% c.i. 1.15–1.96) than the very high-volume centres. A significantly higher risk of mortality associated with low- *versus* very high-volume centres was found in the model before the introduction of the variable ‘urgent surgery’ (before: OR 1.62, 95% c.i. 1.02–2.58, *P* = 0.40; after: OR 1.29, 95% c.i. 0.80–2.07, *P* = 0.3), as shown in *Tables S2, S3*.

**Table 3:** Multivariate analyses evaluating the association between hospital volume and each postoperative outcome recorded*

Hospital volume‡	30-day mortality (*n* = 266)		*P*	Severe complications (*n* = 1670)		*P*
	%	OR (95% c.i.)		%	OR (95% c.i.)	
Very high	1.5	1 (ref. cat.)	–	33.3	1 (ref. cat.)	–
High	1.5	0.93 (0.67–1.28)	0.6	35.3	1.10 (0.94–1.29)	0.2
Medium	1.9	0.84 (0.53–1.33)	0.5	34.4	0.97 (0.78–1.20)	0.8
Low	2.5	1.29 (0.80–2.07)	0.3	42.6	1.50 (1.15–1.96)	0.003

Hospital volume‡	Removal of ≥12 nodes (*n* = 13 497)		*P*	Non-radical resection (R1/2) (*n* = 429)		*P*
	%	OR (95% c.i.)		%	OR (95% c.i.)	
Very high	85.4	1 (ref. cat.)	–	2.9	1 (ref. cat.)	–
High	86.7	1.12 (0.99–1.27)	0.070	2.3	0.92 (0.71–1.19)	0.6
Medium	83.3	0.72 (0.62–0.83)	<0.001	2.2	0.69 (0.48–0.99)	0.045
Low	82.6	0.68 (0.56–0.85)	<0.001	2.0	0.65 (0.40–1.06)	0.09

Hospital volume‡	Neoadjuvant therapy (*n* = 2595)		*P*			
	%	OR (95% c.i.)				
Very high	18.8	1 (ref. cat.)	–			
High	11.6	0.56 (0.48–0.65)	<0.001			
Medium	12.3	0.66 (0.54–0.80)	<0.001			
Low	11.6	0.66 (0.50–0.87)	0.003			

ref. cat.: reference category. *Hospital procedure volume was defined as the number of operations performed during the study period (2017–2022). Low-volume hospitals performed between 19 and 111 procedures; medium-volume hospitals performed between 112 and 167 procedures; high-volume hospitals performed between 168 and 263 procedures; very high-volume hospitals performed between 264 and 638 procedures. †All final models were adjusted for: age, gender, lesion site (rectum *versus* others), aggressive biology, AJCC stage, urgent surgery, history of previous colorectal cancer, history of inflammatory bowel disease, presence of other co-morbidities and familial history of colorectal cancer. Severe complications were classified as Clavien–Dindo grade 3 and higher.

The probability of adequate lymph node sampling was significantly lower in the low-volume (OR 0.68, 95 per cent c.i. 0.56–0.85, *P* < 0.001) and in the medium-volume (OR 0.72, 95 per cent c.i. 0.62–0.83, *P* < 0.001) centres than in the very high-volume centres.

When only cases of rectal cancer were considered (*Table 4*), low-volume centres reported a significantly lower rate of adequate lymph node removal (OR 0.57, 95% c.i. 0.41–0.80, *P* = 0.001) than the very high-volume centres. In the latter centres, patients affected by rectal cancer were more likely to undergo neoadjuvant therapy than those who were treated in high- (OR 0.65, 95% c.i. 0.56–0.77, *P* < 0.001), medium- (OR 0.75, 95% c.i. 0.60–0.92, *P* = 0.006) and low-volume (OR 0.70, 95% c.i. 0.52–0.94, *P* = 0.019) centres, even after adjustment for confounders.

**Table 4:** **Multivariate analyses evaluating the association between hospital volume and each postoperative outcome recorded for patients undergoing surgery for rectal cancer**†

Hospital volume‡	30-day mortality** (*n* = 51)		*P*	Severe complications (*n* = 569)		*P*
	%	OR (95% c.i.)		%	OR (95% c.i.)	
Very high	0.8	1 (ref. cat.)	–	37.7	1 (ref. cat.)	
High	1.1	1.18 (0.56–2.49)	0.7	34.5	0.89 (0.66–1.19)	–
Medium	1.6	1.67 (0.89–3.96)	0.09	43.4	1.27 (0.87–1.87)	0.4
Low	2.2	1.21 (0.86–6.43)	0.09	48.7	1.47 (0.90–2.42)	0.2

Hospital volume‡	Removal of ≥ 12 nodes (*n* = 3042)		*P*	Non-radical resection (R1/2) (*n* = 214)		*P*
	%	OR (95% c.i.)		%	OR (95% c.i.)	
Very high	72.3	1 (ref. cat.)	–	5.1	1 (ref. cat.)	–
High	73.3	1.12 (0.93–1.35)	0.2	4.2	0.77 (0.53–1.12)	0.2
Medium	72.9	0.90 (0.71–1.14)	0.4	3.8	0.54 (0.31–0.94)	0.028
Low	63.5	0.57 (0.41–0.80)	0.001	4.4	0.70 (0.34–1.42)	0.3

Hospital volume‡	Neoadjuvant therapy (*n* = 2331)		*P*			
	%	OR (95% c.i.)				
Very high	54.7	1 (ref. cat.)	–			
High	44.2	0.66 (0.56–0.77)	<0.001			
Medium	43.2	0.75 (0.60–0.92)	0.006			
Low	43.4	0.70 (0.52–0.94)	0.019			

ref. cat.: reference category. *Hospital procedure volume was defined as the number of surgical procedures performed during the study period (2018–2021). Low-volume hospitals performed between 19 and 111 procedures; medium-volume hospitals performed between 112 and 167 procedures; high-volume hospitals performed between 168 and 263 procedures and very high-volume hospitals performed between 264 and 638 procedures. †All final models were adjusted for age, gender, aggressive biology, AJCC stage, urgent surgery, history of previous colorectal cancer, history of inflammatory bowel disease, presence of other co-morbidities and familial history of colorectal cancer. Severe complications were classified as Clavien–Dindo grade 3 and higher. ‡Due to the scarce number of successes (*n* = 51), the final model was adjusted for age, gender, AJCC stage, history of previous colorectal cancer.

The results of the additional analysis of the same outcomes evaluated in the subsample of patients undergoing surgery for rectal cancer when hospital volume was categorized adopting a minimum threshold of ≥10 surgical procedures per year are shown in *Tables S4, S6*.

## Discussion

In this national multicentre study, patients who underwent surgery in a very high-volume centre had a higher chance of adequate lymph node resection. In addition, more frequent utilization of neoadjuvant therapy was observed in the very high-volume centres as compared to all others. Comparing these results to those in the current literature is challenging, as it is unclear^13^ whether low volumes are associated with more frequent postoperative complications and mortality as well as worse pathological/oncological outcomes. A recent meta-analysis showed significant heterogeneity of study populations, location of the cancer and definition of high and low volume^3^. Most studies simply dichotomized the volume based on the median, and the thresholds varied greatly in both the ‘low-’ and ‘high-’ volume centres. In addition, only one study reported the outcomes separately for the colon and the rectum^14^. Of the 47 studies included in the meta-analysis, only 21 reported a statistical adjustment for potential confounders, confirming that the lack of standardization of the methodological quality was a major limitation in many surgical studies^15^.

In the present study, age, gender, AJCC stage and urgent surgery were included in the multivariate analysis *a priori* in order to reduce the bias represented by the expected differences among the centres. Urgent surgery in particular was included because a greater percentage of patients underwent urgent surgery in the low-volume centres (17.5% *versus* 7.6% in the high and very high-volume centres). Interestingly, this resulted in the difference in the rate of mortality between low- and very high-volume centres no longer being statistically significant (from OR 1.62, 95% c.i. 1.02–2.58, to OR 1.29, 95% c.i. 0.80–2.07), indicating the prevailing effect of the timing of surgery on the risk of mortality. Previous studies have shown that perioperative mortality could increase up to 34% when emergency surgery for colorectal cancer was carried out^16^. In the present series, however, urgent surgery was defined as any operation that was performed in the first 48 h after admission. This definition did not strictly refer only to emergency cases, and likely also included those cases that might have been initially treated conservatively, for example by stenting^17^. Moreover, the lack of a dedicated colorectal cancer pathway might have increased the utilization of urgent surgical care and, therefore, increased the risks of suboptimal preoperative assessment of these complex patients. Other significant findings among cases treated in low-volume centres were the inadequate lymph node sampling in both overall (OR 0.68, 95% c.i. 0.56–0.85) and rectal cancer (OR 0.57, 95% c.i. 0.41–0.80) cohorts, as well as the use of neoadjuvant therapy in cases of rectal cancer (OR 0.70, 95% c.i. 0.52–0.94). A similar difference was found when centres were grouped into high- (≥10 cases per year) and low-volume (<10 cases per year) hospitals. While similar findings regarding the association between suboptimal lymph node removal and surgery performed in low-volume centres have been extensively noted in lung, pancreatic and gastric cancer surgery^18–21^, similarly clear evidence regarding colorectal cancer has been lacking^6,22,23^. Although the significance of the number ‘12’ as a threshold has been criticised over the years^24^, it remains one of the most important markers of oncologic adequacy and could reflect not only the expertise of the surgeon but also that of the pathologist^25,26^. This might also explain the reduced rate of neoadjuvant therapy in rectal cancer that was observed in all centres when compared to the very high-volume centres. Similar findings have also been reported by other authors^27,28^.

The present study has a few limitations. Concerning the study design, no long-term follow-up was recorded, and therefore the impact of hospital volume on survival could not be analysed. In addition, the study period partially covered the COVID-19 pandemic, which could represent a source of deviation from the usual outcomes. Moreover, for cases to be included, centres had to voluntarily enrol in the study. This probably excluded many of the smallest centres that are usually included in population-based studies. On the other hand, as proven by the present and other authors’ analyses, the pandemic did not affect the outcomes of colorectal cancer surgery in terms of postoperative complications and adequacy of treatment^8,29^. The voluntary enrolment of the 80 centres provided validated data of a large population of patients treated all over the country in different types of hospitals and collected over a 4-year period, thus counterbalancing the potential variations of volumes and outcomes between years.

A call for the centralization of at least rectal cancer would be a rational answer to these findings. However, drastic variation has been observed regarding the correlation between case volume and outcomes in individual hospitals, as shown by Becerra *et al*. in their study analysing rectal cancer cases from the United States National Cancer Database^27^. Other points should be taken into consideration, namely the possibility of improving colorectal cancer pathways in low-volume centres by implementing multidisciplinary case discussion and audit of outcomes. Moreover, rural or community hospitals with low case volumes could concentrate their colorectal cancer cases at a dedicated local surgical centre in order to increase the volume and improve the outcome.

Although its limitations should be acknowledged, the present study confirmed that hospital volume is strongly associated with the risk of postoperative complications and oncologic adequacy of surgery for colorectal cancer.

## Collaborators

Angela Romano, Angela Belvedere, Antonio Lanci Lanci, Daniele Parlanti, Gabriele Vago, Anna Paola Pezzuto, Anna Canavese, Gerti Dajti, Stefano Cardelli, Caterina Catalioto, Iris S. Russo, Tommaso Violante, Daniele Morezzi, Ludovica Maurino, Eleonora Filippone, Dajana Cuicchi, Paolo Bernante (Surgery of the Alimentary Tract, IRCCS Azienda Ospedaliero-Universitaria di Bologna, Bologna, Italy; Department of Medical and Surgical Sciences, Alma Mater Studiorum University of Bologna, Bologna, Italy); Elio Jovine, Raffaele Lombardi, Michele Masetti, Chiara Cipressi, Maria F. Offi, Cristina Larotonda, Silvana B. Puglisi (Chirurgia A e d'Urgenza IRCCS AOU c/o OM, IRCCS Azienda Ospedaliero-Universitaria di Bologna, Bologna, Italy; Department of Medical and Surgical Sciences, Alma Mater Studiorum University of Bologna, Bologna, Italy); Augusto Barbosa, Roberto Vaiana, Paolo M. Bianchi, Carlo Tonti, Claudio Codignola (Fondazione Poliambulanza Brescia); Luigi Zorcolo, Angelo Restivo, Simona Deidda, Marcello E. Marchetti, Luca Ippolito (Unità Operativa di Chirurgia Coloproctologica—AOU Cagliari); Gaya Spolverato, Salvatore Pucciarelli, Francesco Marchegiani, Giacomo Ghio, Gaia Zagolin, Dajana Glavas, Monica Tomassi (Department of Surgical Oncological and Gastroenterological Sciences, University of Padova—General Surgery 3, Azienda Ospedale Università di Padova); Riccardo Rosati, Ugo Elmore, Lorenzo Gozzini, Riccardo Calef, Francesco Puccetti, Andrea Cossu, Andrea Vignali (Gastrointestinal Surgery Division, IRCCS San Raffaele Hospital, Milan); Mario Morino, Marco E. Allaix, Gaspare Cannata, Erica Lombardi, Carlo A. Ammirati, Chiara Piceni (AOU Città della Salute e della Scienza, Turin, Italy), Piero Buccianti, Riccardo Balestri, Marco Puccini, Daniele Pezzati, Roberto d'Ischia, Vito F. Asta, Benedetta Sargenti, Giacomo Taddei, Federica Bonari, Giulia Boni (Azienda Ospedaliero-Universitaria Pisana); Alessandro Ferrero, Michela Mineccia, Federica Gonella, Marco Palisi, Francesco Danese, Valeria Cherubini, Serena Perotti (Azienda Sanitaria Ospedaliera Ordine Mauriziano Umberto I°, Torino); Michele Carvello, Fabio Carbone, Antonio Luberto, Eleonora Calafiore, Francesca De Lucia, Matteo Sacchi (IRCCS Humanitas Research Hospital, Rozzano, Milan, Italy); Diego Sasia, Maria C. Giuffrida, Edoardo Ballauri, Mathieu Cardile, Serena Armentano, Elsa Beltrami, Gabriele Preve, Barbara Vercellone (Santa Croce and Carle Hospital, Cuneo); Marta Mozzon, Cristina Folliero, Chiara Lirusso, Massimo Vecchiato, Antonio Ziccarelli, Davide Gattesco, Luisa Moretti, Sara Crestale (Chirurgia Generale, Azienda ospedaliero-universitaria S.Maria della Misericordia Udine-ASUFC); Filippo Banchini, Patrizio Capelli, Andrea Romboli, Gerardo Palmieri, Luigi Conti, Nicholas Rizzi (UO Chirurgia Generale Vascolare di Piacenza); Deborah Bonfili (Dipartimento di Chirurgia, Università degli Studi di Parma); Nicolò de Manzini, Paola Germani, Edoardo Osenda, Sara Cortinovis, Carlotta Giunta, Stefano Fracon, Hussein Abdallah, Selene Bogoni (General Surgery Department, University Hospital of Trieste); Nazario Portolani, Riccardo Nascimbeni, Sarah Molfino, Guido A. M. Tiberio, Ilenia Garosio, Giulia Lamperti, Diego Rigosa (UO Chirurgia Generale 3—ASST Spedali Civili Brescia, Università di Brescia); Giorgio Ercolani, Leonardo Solaini, Davide Cavaliere, Andrea Avanzolini, Fabrizio D'Acapito, Leonardo L. Chiarella, Daniela Di Pietrantonio, Domenico Annunziata (Chirurgia generale e TOA, Ospedale Morgagni-Pierantoni, Forlì); Roberta Piccolo, Mario Sorrentino, Mauro Pansini, Alessandro Cojutti, Michele Graziano, Francesco Callegari (UO Chirurgia Generale Ospedale di Latisana-Palmanova, Azienda Ospedaliera Universitaria Friuli Centrale); Laura Balzarotti, Vitale R. Dameno, Antonio Cattaneo, Giuliano Santolamazza, Caterina Altieri, Riccardo Magarini (Ospedale civile ‘G. Fornaroli’, Magenta); Andrea Pietrabissa, Tommaso Dominioni, Luigi Pugliese, Andrea Peri, Marta Botti, Benedetta Sargenti, Francesco Salvetti (Department of Surgery, University of Pavia and Fondazione IRCCS Policlinico San Matteo); Luigi Boni, Elisa Cassinotti, Ludovica Baldari, Valentina Messina, Vera D'Abrosca (SC Chirurgia Generale e Mini-invasiva, Fondazione IRCCS Ca’ Granda Ospedale Maggiore Policlinico—Milano); Pasquale Cianci, Rocco Tumolo, Domenico Gattulli, Enrico Restini, Marina Minafra, Maria Grazia Sederino, Bernardino Bottalico (UOC Chirurgia Generale, Ospedale Lorenzo Bonomo, Andria); Pierluigi Pilati, Boris Franzato, Genny Mattara, Ottavia De Simoni, Andrea Barina, Marco Tonello (Unit of Surgical Oncology of Digestive Tract, Veneto Institute of Oncology IOV-IRCCS, Padua, Italy); Andrea Muratore, Marcello Calabrò, Nicoletta S. Federico Pipitone, Bruno Cuzzola, Elena Herranz Van Nood (Chirurgia Generale Ospedale E. Agnelli, Pinerolo); Nicola Passuello, Alvise Frasson, Enzo Mammano, Luca Faccio, Fabrizio Vittadello, Alice Bressan, Giacomo Sarzo (UOC Chirurgia Generale OSA, ciDIDAS Chirurgia, Azienda Ospedale-Università Padova); Nicolò Tamini, Massimo Oldani, Luca Cigagna, Francesca Carissimi, Giulia De Carlo, Edoardo Baccalini, Luca Nespoli (UO Chirurgia 1, Fondazione IRCCS San Gerardo dei Tintori, Università di Milano-Bicocca); Alessio Giordano, Stefano Cantafio, Lucrezia Grifoni, Davide Matani, Serena Livi (UO di Chirurgia Generale, Nuovo Ospedale ‘S.Stefano’, Azienda ASL Toscana Centro); Daniele Delogu, Fabrizio Scognamillo, Antonio Marrosu, Luca Guerrini (Patologia Chirurgica AOU, Sassari); Giampaolo Ugolini, Federico Ghignone, Giacomo Frascaroli, Nicola Albertini, Davide Zattoni, Giovanni Taffurelli, Isacco Montroni (UO Chirurgia Generale di Ravenna-Faenza, AUSL Romagna); Francesco Colombo, Piergiorgio Danelli, Andrea Bondurri, Anna Maffioli, Alessandro Bonomi, Isabella Pezzoli, Francesco Cammarata (Division of General Surgery—L. Sacco University Hospital, Milano); Orlando Goletti, Mattia Molteni, Alberto Assisi, Giorgio Quartierini (Chirurgia Generale Humanitas Gavazzeni Bergamo, Italy); Corrado Da Lio, Daunia Verdi, Isabella Mondi, Claudia Peluso, Lorenzo Macchi (Department of General Surgery, Mirano Hospital, Venice); Marta Tanzanu, Federico Zanzi, Sara Pellegrini (Chirurgia d'Urgenza, Santa Maria delle Croci—Ravenna); Jacopo Andreuccetti, Rossella D'Alessio, Giusto Pignata, Michele De Capua, Ilaria Canfora, Luca Ottaviani (General Surgery 2, ASST Spedali Civili of Brescia); Pasquale Lepiane, Andrea Balla, Antonio De Carlo, Federica Saraceno, Rosa Scaramuzzo, Anna Guida, Daniele Aguzzi (Ospedale San Paolo, Civitavecchia, Roma); Paolo Bellora, Sergio Gentilli, Manuela Monni, Herald Nikaj (Clinica Chirurgica Ospedale Maggiore della Carità—Novara); Nicola Cillara, Alessandro Cannavera, Antonello Deserra, Carla Margiani, Roberta Cabula (UOC Chirurgia Generale PO Santissima Trinità ASL Cagliari); Manuela Dettori (Oncologia Medica, PO Businco, ARNAS Cagliari); Giulia Gramignano (SSD Oncologia, PO Nostra Signora di Bonaria San Gavino, ASL Medio Campidano); Giovanni Lezoche, Monica Ortenzi, Elena S. Orlandoni, Federica Curzi, Francesca Vitali, Perla Capomagi, Miriam Palmieri (Clinica di Chirurgia Generale e d'urgenza, Ancona Torrette); Mario Giuffrida, Paolo Del Rio, Elena Bonati, Tommaso Loderer, Federico Cozzani, Matteo Rossini, Stefano Agnesi (Clinica Chirurgica Generale—AOU Parma); Gabriella T. Capolupo, Marco Caricato, Filippo Carannante, Gianluca Mascianà, Martina Marrelli, Valentina Miacci, Sara Lauricella (UOC Chirurgia colorettale, Fondazione Policlinico Campus Bio Medico, Roma); Valeria Tonini, Maurizio Cervellera, Salvatore Pisconti, Concetta Lozito, Juliana Shahu, Claudia Mongelli, Giulia Morelli, Lodovico Sartarelli (Ospedale Santissima Annunziata, Taranto); Giuseppe S. Sica, Leandro Siragusa, Giulia Bagaglini, Andrea M. Guida, Marzia Franceschilli, Vittoria Bellato, Cristina Fiorani (Policlinico Tor Vergata, Roma); Antonio Taddei, Matteo Risaliti, Ilenia Bartolini, Maria N. Ringressi, Luca Tirloni (Azienda Ospedaliero Universitaria Careggi, Firenze); Letizia Laface, Emmanuele Abate, Massimiliano Casati, Pietro Gobbi (Ospedale Vittorio Emanuele III Carate Brianza); Enrico Opocher, Nicolò M. Mariani, Andrea Pisani Ceretti, Marco Giovenzana, Beatrice Giuliani, Martina Sironi (ASST Santi Paolo e Carlo, Milano); Ugo Grossi, Giacomo Zanus, Giulio Aniello Santoro, Marco Brizzolari, Eugenio De Leo, Simone Novello, Krizia Aquilino, Francesco Milardi (II Surgery Unit, Regional Hospital Treviso, DISCOG, University of Padua, Italy); Stefano Olmi, Matteo Uccelli, Marta Bonaldi, Giovanni C. Cesana, Marco Bindi (Policlinico San Marco GSD, Zingonia); Raffaele Galleano, Antonio Langone, Massimiliano Botto, Angelo Franceschi, Elena Gambino (Ospedale San Paolo Savona); Maurizio Ronconi, Silvia Casiraghi, Giovanni Casole, Salvatore L. Ciulla (Ospedale di Gardone V.T. - ASST Spedali Civili di Brescia); Giovanni Terrosu, Sergio Calandra, Edoardo Scarpa, Vittorio Cherchi, Giacomo Calini, Lisa Martinuzzo, Lucrezia Clocchiatti, Davide Muschitiello (Clinica Chirurgica, Azienda Sanitaria Universitaria Friuli Centrale ASUFC, Udine); Andrea Romanzi, Barbara Vignati, Alberto Vannelli, Roberta Scolaro, Maria Milanesi, Fabrizio Rossi (Department of General Surgery, Valduce Hospital, Como, Italy); Giuseppe Canonico, Alessandro Anastasi, Tommaso Nelli, Marco Barlettai, Riccardo Fratarcangeli, Carmela Di Martino, Andrea Damigella, Elvira Adinolfi (Ospedale San Giovanni di Dio, Firenze); Arianna Birindelli, Lucio Taglietti, Sara E. Dester (UOC Chirurgia—Ospedale di Esine (BS)—ASST Valcamonica—Italy); Francesco Fleres, Eugenio Cucinotta, Francesca Viscosi, Santino A. Biondo, Giorgio Badessi, Nivia Catarsini, Carmelo Mazzeo (AOU G Martino Policlinico di Messina, Department of General and Emergency Surgery—Italy); Daniela Rega, Paolo Delrio, Carmela Cervone, Alessia Aversano, Silvia De Franciscis, Massimiliano Di Marzo, Bruno Marra, Ugo Pace (Colorectal Surgical Oncology, Department of Abdominal Oncology, Istituto Nazionale Tumori-IRCCS ‘Fondazione G. Pascale’, Naples, Italy); Antonio Amato, Paola Batistotti, Elisa Mina, Alberto Serventi (SC Chirurgia Generale Imperia); Pierfrancesco Lapolla, Andrea Mingoli, Paolo Sapienza, Gioia Brachini, Bruno Cirillo, Enrico Fiori, Daniele Crocetti, Ilaria Clementi (Policlinico Umberto I Sapienza Università di Roma); Gennaro Martines, Arcangelo Picciariello, Giovanni Tomasicchio, Rigers Dibra, Giuseppe Trigiante, Marcella Rinaldi, Giuliano Lantone (Chirurgia Generale ‘M.Rubino’ Azienda Ospedaliero Universitaria Policlinico Bari Italy); Alberto Porcu, Teresa Perra, Antonio M. Scanu, Claudio F. Feo, Alessandro Fancellu, Maria L. Cossu, Giorgio C. Ginesu (Azienda Ospedaliero Universitaria di Sassari, Italia); Alberto Patriti, Diego Coletta, Filippo Petrelli, Paola A. Greco, Claudia Spadoni, Giovanna Cassiani, Federica Bianchini (AO Ospedali Riuniti Marche Nord); Marco Arganini, Matteo Bianchini, Bruno Perotti, Matteo Palmeri (Ospedale Unico della Versilia—Azienda Usl Toscana Nord-ovest); Stefano Scabini, Selene Deiana, Giacomo Carganico, Davide Pertile, Domenico Soriero, Emanuela Fioravanti, Beatrice Sperotto (Unità Operativa Chirurgia Generale ad Indirizzo Oncologico—IRCCS Ospedale Policlinico San Martino, Genova); Bruno Nardo, Daniele Paglione, Veronica Crocco, Marco Doni, Mariasara Osso, Roberto Perri (UOC di Chirurgia Generale ‘Falcone’—Azienda Ospedaliera di Cosenza—Università della Calabria); Gianluca M. Sampietro, Carlo Corbellini, Leonardo Lorusso, Carlo A. Manzo, Maria Cigognini, Caterina Baldi (Division of Surgery, Rho Memorial Hospital—ASST Rhodense—Rho, Milan); Giuseppe Palomba, Giovanni Aprea, Marianna Capuano, Raffaele Basile (AOU Federico II di Napoli—UOC chirurgia endoscopica); Roberta Tutino, Marco Massani, Laura Marinelli, Nicola Canitano (Chirurgia 1—Azienda ULSS2 Marca Trevigiana—Ospedale Regionale di Treviso); Tiziana Pilia, Mauro Podda, Adolfo Pisanu, Valentina Murzi, Silvia Incani, Federica Frongia, Giuseppe Esposito (Policlinico di Monserrato, Chirurgia d'urgenza, Cagliari); Gaetano Luglio, Francesca P. Tropeano, Gianluca Pagano, Eduardo Spina, Giuseppe De Simone, Michele Cricrì (Azienda Ospedaliera Universitaria Federico II); Fausto Catena, Carlo Vallicelli, Nicola Zanini, Diana Ronconi, Francesco Favi, Carlo Mazzucchelli, Girolamo Convertini (Chirurgia Generale e d'Urgenza, Ospedale Bufalini di Cesena, AUSL della Romagna); Leonardo Vincenti, Valeria Andriola, Cinzia Bizzoca (Chirurgia Generale Ospedaliera, Policlinico di Bari); Carlo V. Feo, Nicolò Fabbri, Marta Fazzin, Antonio Pesce, Silvia Gennari, Marco Torchiaro, Silvia Severi (Azienda Unità Sanitaria Locale di Ferrara, Università di Ferrara); Alice Frontali, Greta Bracchetti, Stefano Granieri, Christian Cotsoglou (General Surgery Unit, ASST Vimercate, Vimercate, Italy); Massimo Carlini, Giorgio Lisi, Domenico Spoletini, Maria R. Mastrangeli, Michela Campanelli (UOC Chirurgia Generale, Ospedale Sant'Eugenio, Roma, Italia); Michele Manigrasso, Marco Milone, Giovanni D. De Palma, Sara Vertaldi, Alessia Chini, Francesco Maione, Alessandra Marello (Department of Clinical Medicine and Surgery, ‘Federico II’ University of Naples, Naples, Italy); Francesco Selvaggi, Guido Sciaudone, Lucio Selvaggi, Francesco Menegon Tasselli, Giacomo Fuschillo, Lidia Oddis (Università della Campania Luigi Vanvitelli, Napoli); Simona Grande, Michele Grande (UOSD Chirurgia d’urgenza Tor Vergata); Simona Ascanelli, Laura Chimisso, Filippo Aisoni, Eleonora Rossin, Francesco Pepe, Francesco Marchetti (UO Chirurgia 2 Azienda Ospedaliero-Universitaria Ferrara); Biagio Picardi, Stefano Rossi, Simone Rossi Del Monte, Matteo Picarelli, Irnerio A. Muttillo (Chirurgia Generale e d'Urgenza Ospedale San Filippo Neri ASL Roma 1); Carlo Ratto, Angelo A. Marra, Angelo Parello, Francesco Litta, Paola Campennì, Veronica De Simone (Fondazione Policlinico Universitario Agostino Gemelli, IRCCS, Roma); Francesco Pata (Department of Surgery, Nicola Giannettasio Hospital, Corigliano-Rossano, Italy; La Sapienza University, Rome, Italy); Cristiana Riboni (EOC Ospedale Regionale di Lugano, Lugano, Switzerland); Emanuele Rausa (Unit of Hereditary Digestive Tumours, Fondazione IRCCS-National Cancer Institute, Milan, Italy); Valerio Celentano (Chelsea and Westminster Hospital NHS Foundation Trust, London, UK; Department of Surgery and Cancer, Imperial College, London, UK).

## Supplementary Material

znad373_Supplementary_Data

## Data Availability

The data will be made available upon request to the corresponding author. The results of this study were presented as an oral communication at the 29th annual meeting of the European Surgical Association (ESA) in Bordeaux, 12–13 May 2023.
