# Supplementary material for: Effect of centre volume on pathological outcomes and postoperative complications after surgery for colorectal cancer: results of a multicentre national study
Source: Br J Surg. 2023 Nov 14;111(1):znad373. doi: 10.1093/bjs/znad373 (PMC10771132; doi:10.1093/bjs/znad373)
Supplement: znad373_Supplementary_Data [file znad373_supplementary_data.docx]

**Title: Effect of centre volume on pathological outcomes and postoperative complications after surgery for colorectal cancer: results of a multicentre national study**

Matteo Rottoli,^1,2^ Antonino Spinelli,^3,4^ Gianluca Pellino,^5,6^ Alice Gori,^1,2^ Giacomo Calini,^1,2^ Maria E Flacco,^7^ Lamberto Manzoli,^2^ Gilberto Poggioli;^1,2^ and the COVID-CRC Study Group.

Members of the COVID-CRC Study Group are co-authors of this study and are listed under the heading Collaborators

^1^ Surgery of the Alimentary Tract, IRCCS Azienda Ospedaliero-Universitaria di Bologna, Bologna, Italy

^2^ Department of Medical and Surgical Sciences, Alma Mater Studiorum University of Bologna, Bologna, Italy

^3^ Department of Biomedical Sciences, Humanitas University, Pieve Emanuele, Milan, Italy

^4^ IRCCS Humanitas Research Hospital, Rozzano, Milan, Italy

^5^ Department of Advanced Medical and Surgical Sciences, Università degli Studi della Campania Luigi Vanvitelli, Naples, Italy

^6^ Colorectal Surgery, University Hospital Vall d’Hebron, Barcelona, Spain

^7^ Department of Environmental and Preventive Sciences, University of Ferrara, Ferrara, Italy

Corresponding author:

Matteo Rottoli, MD, PhD

Surgery of the Alimentary Tract, IRCCS Azienda Ospedaliero Universitaria di Bologna.

Department of Medical and Surgical Sciences, Alma Mater Studiorum University of Bologna.

Via Massarenti 9 - 40138 Bologna, Italy

ORCID ID: 0000-0003-0278-4139

Telephone number: +390512145262

Email: matteo.rottoli2@unibo.it

**Supplementary Materials - Index**

| **Supplementary Figures and Tables** |  |
| --- | --- |
| Supplementary Table 1 | *Page 3* |
| Supplementary Table 2 | *Page 4* |
| Supplementary Table 3 | *Page 5* |
| Supplementary Table 4 | *Page 6* |
| Supplementary Table 5 | *Page 8* |
| Supplementary Table 6 | *Page 10* |
|  |  |
|  |  |

**Supplementary Table 1.**

**Centres grouped according to hospital volume quartiles within the 4-years of the study (2018-2021)**

| Group | Number of procedures (4 years) | Overall number of patients | Number of centers |
| --- | --- | --- | --- |
| LOW | 19-111 | 999 | 21 |
| MEDIUM | 112-167 | 2644 | 19 |
| HIGH | 168-263 | 4675 | 20 |
| VERY-HIGH | ≥264 | 8565 | 20 |

**Supplementary Table 2 - Results of the multivariate analysis evaluating the association between hospital volume with postoperative outcomes (2A) and pathological and oncological outcomes (2B) in the overall cohort when not adjusted for urgent surgery**

**Supplementary Table 2A**.

|  | **30-day mortality**  (n=266) | |  | **Severe complications**  (n=1670) | |  |
| --- | --- | --- | --- | --- | --- | --- |
| Hospital volume **^‡^** | % | OR (95% CI) | p | % | OR (95% CI) | p |
|  |  |  |  |  |  |  |
| Very high | 1.5 | 1 (ref. cat.) | -- | 33.3 | 1 (ref. cat.) | -- |
| High | 1.5 | 0.94 (0.68-1.30) | 0.7 | 35.3 | 1.10 (0.94-1.29) | 0.2 |
| Medium | 1.9 | 0.87 (0.55-1.37) | 0.5 | 34.4 | 0.97 (0.78-1.20) | 0.8 |
| Low | 2.5 | 1.62 (1.02-2.58) | 0.040 | 42.6 | 3.28 (1.19-2.02) | 0.001 |
|  |  |  |  |  |  |  |

**Supplementary Table 2B.** Overall - Multivariate analyses evaluating the association between hospital volume, and each pathological and oncologic outcome recorded *.

|  | **Removal of ≥12 nodes**  (n=13,497) | |  | **Non-radical resection**  (n=429) | |  | **Neoadjuvant therapy**  (n=2595) | |  |
| --- | --- | --- | --- | --- | --- | --- | --- | --- | --- |
| Hospital volume **^‡^** | % | OR (95% CI) | p | % | OR (95% CI) | p | % | OR (95% CI) | p |
|  |  |  |  |  |  |  |  |  |  |
| Very high | 85.4 | 1 (ref. cat.) | -- | 2.9 | 1 (ref. cat.) | -- | 18.8 | 1 (ref. cat.) | -- |
| High | 86.7 | 1.12 (0.99-1.27) | 0.070 | 2.3 | 0.94 80.73-1.21) | 0.6 | 11.6 | 0.55 (0.47-0.64) | <0.001 |
| Medium | 83.3 | 0.72 (0.62-0.83) | <0.001 | 2.2 | 0.71 (0.49-1.01) | 0.06 | 12.3 | 0.63 (0.52-0.77) | <0.001 |
| Low | 82.6 | 0.68 (0.56-0.84) | <0.001 | 2.0 | 0.74 (0.46-1.20) | 0.2 | 11.6 | 0.62 (0.47-0.81) | <0.001 |
|  |  |  |  |  |  |  |  |  |  |

**^‡^** Hospital procedure volume was defined as the number of surgical procedures performed during the study period (2018-2021). Low-volume hospitals performed between 19 and 111 procedures; medium-volume hospitals performed between 112 and 167 procedures; high-volume hospitals performed between 168 and 263 procedures; very high-volume hospitals performed between 264 and 638 procedures.

* All final models were adjusted for: age, gender, lesion site (rectum vs. others), aggressive biology, AJCC stage, history of previous colorectal cancer, history of inflammatory bowel disease, presence of other comorbidities and familial history of colorectal cancer. Severe complications were classified as Clavien-Dindo grade 3 and higher.

OR: odds ratio; CI: confidence interval; ref. cat.: reference category; AJCC: American Joint Committee on Cancer

**Supplementary Table 3 - Results of the multivariate analysis evaluating the association between hospital volume with postoperative outcomes (3A) and pathological and oncological outcomes (3B) in the rectal cancer cohort when not adjusted for urgent surgery**

**Supplementary Table 3A**.

|  | **30-day mortality****  (n=51) | |  | **Severe complications**  (n=569) | |  |
| --- | --- | --- | --- | --- | --- | --- |
| Hospital volume **^‡^** | % | OR (95% CI) | p | % | OR (95% CI) | p |
|  |  |  |  |  |  |  |
| Very high | 0.8 | 1 (ref. cat.) | -- | 37.7 | 1 (ref. cat.) | -- |
| High | 1.1 | 1.31 (0.65-2.63) | 0.5 | 34.5 | 0.89 (0.66-1.19) | 0.4 |
| Medium | 1.6 | 1.67 (0.89-3.96) | 0.09 | 43.4 | 1.28 (0.87-1.87) | 0.2 |
| Low | 2.2 | 1.21 (0.86-6.43) | 0.09 | 48.7 | 1.48 (0.91-2.83) | 0.12 |
|  |  |  |  |  |  |  |

**Supplementary Table 3B.**

|  | **Removal of ≥12 nodes**  (n=3042) | |  | **Non-radical resection**  (n=214) | |  | **Neoadjuvant therapy**  (n=2331) | |  |
| --- | --- | --- | --- | --- | --- | --- | --- | --- | --- |
| Hospital volume **^‡^** | % | OR (95% CI) | p | % | OR (95% CI) | p | % | OR (95% CI) | p |
|  |  |  |  |  |  |  |  |  |  |
| Very high | 72.3 | 1 (ref. cat.) | -- | 5.1 | 1 (ref. cat.) | -- | 54.7 | 1 (ref. cat.) | -- |
| High | 73.3 | 1.13 (0.93-1.36) | 0.2 | 4.2 | 0.79 (0.54-1.15) | 0.2 | 44.2 | 0.65 (0.55-0.76) | <0.001 |
| Medium | 72.9 | 0.90 (0.71-1.15) | 0.4 | 3.8 | 0.56 (0.32-0.98) | 0.01 | 43.2 | 0.72 (0.58-0.88) | 0.002 |
| Low | 63.5 | 0.58 (0.42-0.81) | 0.01 | 4.4 | 0.78 (0.39-1.57) | 0.5 | 43.4 | 0.66 (0.49-0.89) | 0.006 |
|  |  |  |  |  |  |  |  |  |  |

**^‡^** Hospital procedure volume was defined as the number of operations performed during the study period (2018-2021). Low-volume hospitals performed between 19 and 111 procedures; medium-volume hospitals performed between 112 and 167 procedures; high-volume hospitals performed between 168 and 263 procedures and very high-volume hospitals performed between 264 and 638 procedures.

* All final models were adjusted for age, gender, aggressive biology, AJCC stage, history of previous colorectal cancer, history of inflammatory bowel disease, presence of other comorbidities and familial history of colorectal cancer. Severe complications were classified as Clavien-Dindo grade 3 and higher.

** Due to the scarce number of successes (n=51), the final model was adjusted for age, gender, AJCC stage, history of previous colorectal cancer.

OR: odds ratio; CI: confidence interval; ref. cat.: reference category.

**Supplementary Table 4**. Selected demographic and clinical characteristics and outcomes, overall and by hospital surgical volume (**^‡^** high volume: ≥10 surgery cases per year; low volume: <10 surgery cases per year) in the sub-sample of patients with rectal cancer.

|  | **Overall sample** | **Low volume^‡^** | **High volume^‡^** |  |
| --- | --- | --- | --- | --- |
| Patients (hospitals), n | 4676 (79)* | 869 (39) | 3807 (40) | **p **** |
|  |  |  |  |  |
| Male gender, % | 39.1 | 40.6 | 38.8 | 0.3 |
|  |  |  |  |  |
| Mean age at diagnosis in years (SD) | 67.8 (12.3) | 69.6 (11.7) | 67.4 (12.4) | <0.001 |
|  |  |  |  |  |
| Mean BMI (SD) | 25.3 (4.3) | 25.3 (4.0) | 25.3 (4.0) | 0.9 |
|  |  |  |  |  |
| Current or past smoker, % | 44.9 | 42.8 | 45.4 | 0.2 |
|  |  |  |  |  |
| Surgery performed during the pandemic period, % | 42.6 | 47.4 | 41.6 | 0.02 |
|  |  |  |  |  |
| *Comorbidities:* |  |  |  |  |
| Family history of colorectal cancer, % | 11.7 | 9.4 | 12.2 | 0.04 |
|  |  |  |  |  |
| Previous history of colorectal cancer, % | 2.3 | 0.8 | 2.7 | 0.001 |
|  |  |  |  |  |
| Previous history of cancer (others), % | 10.4 | 8.6 | 10.5 | 0.06 |
|  |  |  |  |  |
| Inflammatory bowel disease, % | 1.15 | 1.15 | 1.16 | 0.9 |
|  |  |  |  |  |
| Other comorbidities, % |  |  |  | 0.6 |
| - None | 26.9 | 27.5 | 26.8 |  |
| - 1 | 41.0 | 42.0 | 41.0 |  |
| - 2 | 22.8 | 22.2 | 22.9 |  |
| - 3 or more | 9.3 | 8.3 | 9.6 |  |
|  |  |  |  |  |
| Previous polypectomy, % | 1.5 | 1.3 | 1.5 | 0.6 |
|  |  |  |  |  |
| Asymptomatic disease, % | 15.2 | 14.5 | 15.3 | 0.5 |
|  |  |  |  |  |
|  | (n=4510) | (n=782) | (n=3728) |  |
| Fecal occult blood test performed, % | 20.8 | 20.6 | 20.3 | 0.9 |
|  |  |  |  |  |
| T4 stage, % | 6.9 | 6.9 | 6.9 | 0.9 |
|  |  |  |  |  |
|  | (n=4326) | (n=740) | (n=3586) |  |
| Synchronous adenomas, % | 19.8 | 16.8 | 20.4 | 0.03 |
|  |  |  |  |  |
|  | (n=4330) | (n=771) | (n=3559) |  |
| Synchronous cancers, % | 3.7 | 3.5 | 3.7 | 0.8 |
|  |  |  |  |  |
| Metastatic lesions, % | 10.9 | 11.4 | 10.8 | 0.6 |
|  |  |  |  |  |
| Stenosing lesions, % | 8.6 | 9.6 | 8.3 | 0.2 |
|  |  |  |  |  |
| Urgent surgery, % | 4.2 | 7.9 | 3.3 | <0.001 |
|  |  |  |  |  |
|  | (N=4257) | (N=783) | (N=3474) |  |
| Mean serum Hb, g/dL (SD) | 128.2 (18.5) | 123.4 (19.0) | 129.3 (18.2) | <0.001 |
|  |  |  |  |  |
|  | (N=4057) | (N=687) | (N=3370) |  |
| Mean blood glucose, mg/dL (SD) | 105.5 (29.0) | 108.8 (33.8) | 104.9 (27.9) | 0.001 |
|  |  |  |  |  |
|  | (N=3021) | (N=459) | (N=2562) |  |
| Median CEA, ng/mL (IQR) | 2.8 (4.8) | 3.0 (6.0) | 2.4 (5.0) | <0.001 |
|  |  |  |  |  |
| ASA score >2, % | 37.9 | 42.1 | 37.0 | 0.005 |
|  |  |  |  |  |
|  | (N=2976) | (N=663) | (N=2313) |  |
| Anastomosis, % | 88.3 | 86.4 | 88.9 | 0.09 |
|  |  |  |  |  |
| Additional surgery, % | 10.5 | 10.1 | 10.5 | 0.7 |
|  |  |  |  |  |
| Laparoscopic surgery, % | 74.0 | 64.0 | 76.2 | <0.001 |
|  |  |  |  |  |
|  | (N=3442) | (N=554) | (N=2888) |  |
| Conversion to open surgery, % | 6.7 | 7.6 | 6.5 | 0.3 |
|  |  |  |  |  |
|  | (N=3560) | (N=672) | (N=2888) |  |
| Loop ileostomy, % | 56.9 | 49.1 | 58.7 | <0.001 |
|  |  |  |  |  |
| ICU admission, % | 14.2 | 13.7 | 14.3 | 0.7 |
|  |  |  |  |  |

* 1 Unit reported zero cases of rectal cancer surgery. ** Chi-squared test for categorical variables; t-test and Kruskal-Wallis test for parametric and non-parametric continuous variables, respectively.

SD: standard deviation; IQR: interquartile range; BMI: Body Mass Index; CEA: carcinoembryonic antigen; ASA: American Society of Anesthesiologists; ICU: Intensive Care Unit.

**Supplementary Table 5**. Pathological and postoperative variables and outcomes, overall and by hospital surgical volume (**^‡^** high volume: ≥10 surgery cases per year; low volume: <10 surgery cases per year) in the sub-sample of patients with rectal cancer.

|  | **Overall sample** | **Low volume^‡^** | **High volume^‡^** |  |
| --- | --- | --- | --- | --- |
| Patients (hospitals), n | 4676 (79)* | 869 (39) | 3807 (40) | **p **** |
|  |  |  |  |  |
| Multiple lesions, % | 4.2 | 3.9 | 4.2 | 0.7 |
|  |  |  |  |  |
|  | (N=4235) | (N=826) | (N=3409) |  |
| Aggressive biology, % | 60.9 | 59.2 | 60.3 | 0.3 |
|  |  |  |  |  |
| Histology, % |  |  |  |  |
| - Adenocarcinoma | 96.3 | 96.4 | 96.3 | 0.9 |
|  |  |  |  |  |
| AJCC stage, % | (N=4235) | (N=826) | (N=3409) |  |
| - 0 | 3.4 | 3.2 | 3.5 | 0.3 |
| - I | 32.5 | 26.4 | 34.0 | <0.001 |
| - II | 24.0 | 28.0 | 23.0 | 0.003 |
| - III | 28.0 | 30.4 | 27.4 | 0.02 |
| - IV | 12.1 | 12.0 | 12.1 | 0.9 |
|  |  |  |  |  |
| Median in-hospital length of stay (IQR) | 8.0 (6.0) | 8.0 (6.0) | 8.0 (6.0) | 0.9 |
|  |  |  |  |  |
| *Postoperative medical complications, %:* |  |  |  |  |
| All complications ^E^ | 16.2 | 18.2 | 15.7 | 0.07 |
| Anemia | 3.7 | 6.1 | 3.2 | <0.001 |
| Pulmonary complications ^F^ | 2.7 | 3.3 | 2.6 | 0.2 |
| Sepsis | 2.1 | 2.3 | 2.1 | 0.3 |
| Acute kidney failure | 1.3 | 1.6 | 1.2 | 0.4 |
| Myocardial infarction | 0.36 | 0.81 | 0.26 | 0.02 |
| Venous thromboembolism | 0.32 | 0.23 | 0.34 | 0.6 |
| Pulmonary embolism | 0.21 | 0.12 | 0.24 | 0.5 |
| Stroke | 0.11 | 0.12 | 0.11 | 0.9 |
|  |  |  |  |  |
| *Postoperative surgical complications, %:* |  |  |  |  |
| All complications ^G^ | 21.1 | 19.5 | 21.4 | 0.2 |
| Surgical site infection | 4.2 | 4.7 | 4.0 | 0.4 |
| Intra-abdominal bleeding | 1.1 | 1.4 | 1.1 | 0.4 |
| Intra-luminal bleeding | 0.66 | 0.69 | 0.66 | 0.9 |
| Intra-abdominal sepsis ^H^ | 9.2 | 8.6 | 9.3 | 0.5 |
| Dehiscence | 7.6 | 7.7 | 7.6 | 0.9 |
| Abdominal abscess | 3.9 | 2.3 | 4.3 | 0.007 |
| Peritonitis | 0.81 | 0.46 | 0.89 | 0.2 |
| Paralitic ileus | 2.9 | 2.2 | 3.1 | 0.2 |
| Bowel occlusion | 1.5 | 1.0 | 1.7 | 0.2 |
|  |  |  |  |  |
| *Outcomes:* |  |  |  |  |
|  |  |  |  |  |
| 30-day mortality, % | 1.1 | 2.1 | 0.87 | 0.002 |
|  |  |  |  |  |
|  | (N=1484) | (N=274) | (N=1210) |  |
| Clavien-Dindo classification ≥3, % | 38.3 | 39.8 | 38.0 | 0.6 |
|  |  |  |  |  |
| *Number of resected lymph nodes:* | (N=4213) | (N=804) | (N=3409) |  |
| - Median (IQR) | 15.0 (10.0) | 14.0 (10.0) | 15.0 (11.0) | <0.001 |
| - ≥12 lymph nodes, % | 72.2 | 67.9 | 73.2 | 0.033 |
|  |  |  |  |  |
| Non- radical surgery, % | 4.7 | 4.8 | 4.6 | 0.8 |
|  |  |  |  |  |
| Neoadjuvant therapy, % | 49.9 | 40.6 | 52.0 | <0.001 |
|  |  |  |  |  |

* 1 Unit reported zero cases of rectal cancer surgery. ** Chi-squared test for categorical variables; t-test and Kruskal-Wallis test for parametric and non-parametric continuous variables, respectively.

^E^ Including: anemia, myocardial infarction, stroke, pulmonary embolism, venous thromboembolism, acute kidney failure, sepsis, pneumonia, acute respiratory distress syndrome, respiratory failure.

^F^ Pneumonia, and/or acute respiratory distress syndrome, and/or respiratory failure.

^G^ Including intra-abdominal or intra-luminal bleeding, dehiscence, surgical site infection, abdominal abscess, peritonitis, paralitic ileus, bowel occlusion.

^H^ Including dehiscence, abdominal abscess, and peritonitis.

SD: standard deviation; IQR: interquartile range; AJCC: American Joint Committee on Cancer.

**Supplementary Table 6 - Results of the multivariate analysis evaluating the association between hospital volume with postoperative outcomes (6A) and pathological and oncological outcomes (6B) in the rectal cancer cohort**

**Supplementary Table 6A**.

|  | **30-day mortality****  (n=51) | |  | **Severe complications**  (n=569) | |  |
| --- | --- | --- | --- | --- | --- | --- |
| Hospital volume **^‡^** | % | OR (95% CI) | p | % | OR (95% CI) | p |
|  |  |  |  |  |  |  |
| High | 0.87 | 1 (ref. cat.) | -- | 38.0 | 1 (ref. cat.) | -- |
| Low | 2.07 | 1.73 (0.84-3.55) | 0.13 | 39.8 | 1.15 (0.84-1.56) | 0.4 |
|  |  |  |  |  |  |  |

**Supplementary Table 6B.**

|  | **Removal of ≥12 nodes**  (n=3042) | |  | **Non-radical resection**  (n=214) | |  | **Neoadjuvant therapy**  (n=2331) | |  |
| --- | --- | --- | --- | --- | --- | --- | --- | --- | --- |
| Hospital volume **^‡^** | % | OR (95% CI) | p | % | OR (95% CI) | p | % | OR (95% CI) | p |
|  |  |  |  |  |  |  |  |  |  |
| High | 73.2 | 1 (ref. cat.) | -- | 4.6 | 1 (ref. cat.) | -- | 52.0 | 1 (ref. cat.) | -- |
| Low | 67.9 | 0.65 (0.54-0.80) | <0.001 | 4.8 | 0.77 (0.50-1.18) | 0.2 | 40.6 | 0.64 (0.54-0.77) | <0.001 |
|  |  |  |  |  |  |  |  |  |  |

**^‡^** Hospital procedure volume was defined as the number of surgical procedures performed during the study period (2018-2021). High volume: ≥10 surgery cases per year; low volume: <10 surgery cases per year.

* All final models were adjusted for age, gender, aggressive biology, AJCC stage, urgent surgery, history of previous colorectal cancer, history of inflammatory bowel disease, presence of other comorbidities and familial history of colorectal cancer. Severe complications were classified as Clavien-Dindo grade 3 and higher.

** Due to the scarce number of successes (n=51), the final model was adjusted for age, gender, AJCC stage, history of previous colorectal cancer.

OR: odds ratio; CI: confidence interval; ref. cat.: reference category.
